# KANPHOS: Kinase-associated neural phospho-signaling database for data-driven research

**DOI:** 10.3389/fnmol.2024.1379089

**Published:** 2024-04-02

**Authors:** Takayuki Kannon, Satoshi Murashige, Tomoki Nishioka, Mutsuki Amano, Yasuhiro Funahashi, Daisuke Tsuboi, Yukie Yamahashi, Taku Nagai, Kozo Kaibuchi, Junichiro Yoshimoto

**Affiliations:** ^1^Department of Biomedical Data Science, Fujita Health University School of Medicine, Toyoake, Japan; ^2^Division of Computational Science, International Center for Brain Science, Fujita Health University, Toyoake, Japan; ^3^Division of Cell Biology, International Center for Brain Science, Fujita Health University, Toyoake, Japan; ^4^Department of Cell Pharmacology, Graduate School of Medicine, Nagoya University, Nagoya, Japan; ^5^Division of Behavioral Neuropharmacology, International Center for Brain Science, Fujita Health University, Toyoake, Japan

**Keywords:** database, protein phosphorylation, data-driven research, pathway analysis, Kinase-Associated Neural Signaling

## Abstract

Protein phosphorylation, a key regulator of cellular processes, plays a central role in brain function and is implicated in neurological disorders. Information on protein phosphorylation is expected to be a clue for understanding various neuropsychiatric disorders and developing therapeutic strategies. Nonetheless, existing databases lack a specific focus on phosphorylation events in the brain, which are crucial for investigating the downstream pathway regulated by neurotransmitters. To overcome the gap, we have developed a web-based database named “Kinase-Associated Neural PHOspho-Signaling (KANPHOS).” This paper presents the design concept, detailed features, and a series of improvements for KANPHOS. KANPHOS is designed to support data-driven research by fulfilling three key objectives: (1) enabling the search for protein kinases and their substrates related to extracellular signals or diseases; (2) facilitating a consolidated search for information encompassing phosphorylated substrate genes, proteins, mutant mice, diseases, and more; and (3) offering integrated functionalities to support pathway and network analysis. KANPHOS is also equipped with API functionality to interact with external databases and analysis tools, enhancing its utility in data-driven investigations. Those key features represent a critical step toward unraveling the complex landscape of protein phosphorylation in the brain, with implications for elucidating the molecular mechanisms underlying neurological disorders. KANPHOS is freely accessible to all researchers at https://kanphos.jp.

## 1 Introduction

Protein phosphorylation is pivotal in regulating cellular processes such as signal transduction, cell cycle control, and metabolic pathways (Cohen, 2000). In the brain, protein phosphorylation, through its ability to modulate protein function and signaling pathways, is central to various neuronal functions such as synaptic plasticity, neurotransmitter release, and neuronal development (Lisman, 2003; Sweatt, 2004). Dysregulation of protein phosphorylation has been implicated in numerous neurological disorders, including Alzheimer’s disease, Parkinson’s disease, and schizophrenia, making it a focal point for research aimed at understanding the molecular underpinnings of these conditions (Cohen, 2001; Coba et al., 2009; McGuire et al., 2017).

With advancements in genomics and mass spectrometry (MS)-based proteomics, over 500 protein kinases have been found to be encoded in the human genome (Manning et al., 2002), and a large number of phosphorylated sites of proteins have been reported from mouse and human samples (Ballif et al., 2004; Beausoleil et al., 2004; Hornbeck et al., 2012). Databases comprehensively collecting combinations of protein kinases and their phosphorylated sites are beneficial to estimating potential (but unidentified) pathways, thereby exploring/predicting key factors to regulate biological functions.

The scientific community has recognized the importance of such databases, with several notable efforts in this direction—for example, PhosphoSitePlus (Hornbeck et al., 2012) and Phospho.ELM (Dinkel et al., 2011) has made significant contributions to cataloging phosphorylation events and kinase–substrate relationships. These resources have been invaluable for researchers studying signal transduction pathways, kinome-wide analyses, and the functional implications of protein phosphorylation.

However, none of these existing databases focus on phosphorylation events in the brain, which are crucial for investigating the downstream pathway regulated by neurotransmitters. To overcome the limitation, we developed the kinase-interacting substrate screening (KISS) (Nishioka et al., 2012; Amano et al., 2015; Nagai et al., 2016) method for *in vitro* phosphoproteins and the kinase-oriented substrate screening (KIOSS) (Nishioka et al., 2012; Nagai et al., 2016; Funahashi et al., 2020; Yamahashi et al., 2022) method for *in vivo* phosphoproteins. For informatics infrastructure, we are also developing a web-based database named “Kinase-Associated Neural PHOspho-Signaling (KANPHOS)” to widely provide accumulated information on protein phosphorylation as a basis for data-driven research. Using KANPHOS, we can systematically browse the database for information on upstream signals such as neurotransmitters, kinases, phosphorylated substrates, phosphorylation site positions, amino acid sequences, and associated metadata in the database.

The former version of KANPHOS was based on a content management system (CMS) called XooNIps (Yamaji et al., 2007; Ahammad et al., 2021). The advantage of using XooNIps at the time was that it was easy to build systems, extend functionality, and change designs. This was very useful for quickly building a database with feedback from researchers and making improvements. However, XooNIps was originally a rich document-oriented archive database that handled various data types, such as videos and images. This led to a significant overhead for each data point, and the speed decreased when handling a large amount of fine-grained data such as KANPHOS. Furthermore, there was a problem with server overload because the server used a server-side rendering method that processed a large amount of information on the server side before displaying it in the browser. In addition, it was an application programming interface (API) specialized in cross-search that connected several databases, so it took considerable work to collaborate using analysis tools. To address those technical issues, we have rebuilt the system as the next-generation version of KANPHOS, based on React^1^ and Django REST Framework.^2^

This paper presents the design concept, detailed features, and improvement report of KANPHOS. This publicly accessible web-based database provides a comprehensive collection of information on protein phosphorylation for data-driven research.

## 2 Materials and methods

### 2.1 Design principles of KANPHOS

To clarify the significance of databases in life science, we begin with the basic workflow of data-driven research.

Data-driven research uses data collected from experiments or observations to generate hypotheses, test them, and gain new insights. The data-driven research cycle consists of four steps:

Data collection: a step to collect data relevant to the phenomenon or system under study. Data collection methods include experimentation, observation, and literature review.

Data analysis: a step to analyze the collected data to identify patterns and trends. Data analysis techniques include statistics, machine learning, and other methods.

Hypothesis generation: a step to generate hypotheses based on the results of data analysis. Hypotheses serve as a guide for understanding the phenomenon or system.

Hypothesis testing: a step to test/validate hypotheses through new experimentation or observation. If the hypothesis is supported, new knowledge is gained.

Databases play an important role in data-driven research. Databases facilitate efficient data collection, storage, retrieval, and analysis. Databases make it easier to share and reuse data, which can lead to research efficiency and productivity improvements.

**Figure 1:**
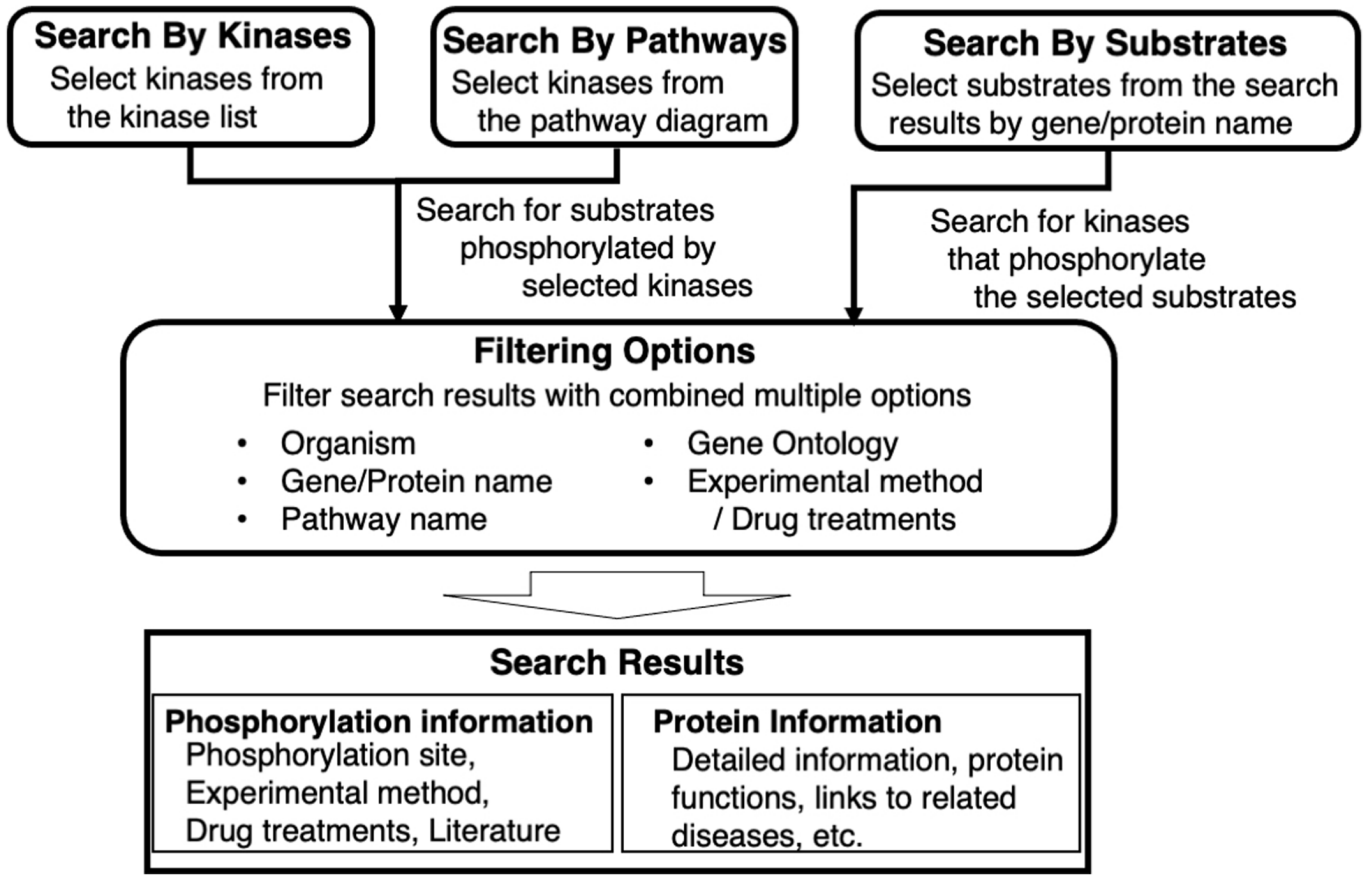
Overview of key features of KANPHOS. KANPHOS supports three search modes: “Search by Kinases” shows us the list of substrates phosphorylated by specific kinases; “Search by Pathways” helps us to select a kinase of interest from pathway diagrams, followed by showing the list of substrates phosphorylated by the kinase; and “Search by Substrates” shows the list of kinases phosphorylating proteins of interest. Search results can be narrowed down by combining multiple items, and two types of search results are displayed for each result: phosphorylation information and protein information.

KANPHOS is a database designed to satisfy the following three requirements for supporting data-driven research on protein phosphorylation (see also Figure 1):

1. To search for protein kinases and their phosphorylated substrates for each extracellular signal or disease.
2. To realize a one-stop search for information related to phosphorylated substrate genes, proteins, mutant mice, diseases, etc.
3. To include a linkage function supporting pathway and network analysis.

The search feature is divided into three elements. One is to search for phosphorylated substrates by a selected kinase from a list. The second is to search for substrates that are phosphorylated in the same way by selecting a kinase from a signaling pathway diagram or pathway map. The third is to search for protein kinases that phosphorylate a selected substrate from a gene or protein name. The search results can be filtered by combining multiple items, including animal species, gene/protein name, pathway name, Gene Ontology, experimental method, and used drugs. The displayed search results provide two types of information for each result. One is experimental information, such as the phosphorylation site, experimental method, drugs used, and links to literature information. The other is related information, such as details of protein function and links to related diseases.

### 2.2 Data models

Our research group has developed a comprehensive dataset of phosphorylated substrates for major kinases using the KISS method for *in vitro* identification and the KIOSS method for *in vivo* identification. We also have a dataset that comprehensively identifies phosphorylated substrates and phosphorylation sites linked to upstream signals activated by neurotransmitters such as dopamine, acetylcholine (Ach), and adenosine. KANPHOS was developed to systematically search these data by linking them as relational data. KANPHOS handles these data as two data models: one for phosphorylation and another for protein information.

#### 2.2.1 Phosphorylation information

This data model stores the relationship between a phosphorylation substrate and a kinase. The data include information such as the kinase, substrate, phosphorylation site, and experimental method (Table 1). Each phosphorylation site is stored as a single entry per experiment. Record_ID is a unique identifier for entries. The kinase information includes the UniProtKB accession number (AC), kinase name, subtype, and family name. The substrate information includes the UniProtKB AC and substrate name. The phosphorylation site information includes the amino acid residue, site position, and surrounding sequence. The experiment information includes a description of the experiment, the experimental method, the experimental conditions (*in vivo* or *in vitro*, extracellular signals, and associated drug treatments), and citations to the literature that support the data. The entry also includes information about the user group allowed to access the entry. This information can be used to restrict access to data that still needs to be published or is under curation.

**Table 1:** Field names of phosphorylation information entries stored in KANPHOS.

Field name	Description
Record_ID	Identifier of phosphorylation information entry
PermittedGroup	Groups permitted to view the entry
EnzymeAccNo	UniProtKB accession number of kinase protein
KinaseName	Gene symbol of kinase protein
KinaseSubType	Subtype name of kinase protein
KinaseFamily	Family name of the kinase protein
SubstrateAccNo	UniProtKB accession number of substrate protein
SubstrateName	Gene symbol of substrate protein
AminoAcid	Amino acid residues of the substrate protein
SiteNo	Position of the phosphorylation site
SeqWindow	Sequence around the phosphorylation site
Method	Experimental method
SignalName	Extracellular signals
VivoVitro	*In Vivo* or *In Vitro*
Treatments	Associated drug treatments
Description	Description of the experiment
Refs	Referenced published papers
PMIDs	PubMed Identifiers of referenced published papers

#### 2.2.2 Protein information

KANPHOS stores protein information for each kinase and substrate that is the subject of the search (Table 2). The primary ID of each protein information entry is the same as the UniProt Knowledgebase accession number (UniProtKB AC) (UniProt Consortium, 2023). UniProtKB AC is an ID uniquely assigned to each protein entry registered in the UniProtKB, which collects information on protein functions. Even if multiple entries are merged or deleted, all associated UniProtKB ACs are maintained. KANPHOS uses UniProtKB AC because it refers to protein names, gene names and their synonyms, animal species, and summary articles of functions from UniProt. KANPHOS also has the ID of the protein information entry used by each database to link to various databases that summarize information about proteins. Currently, the linked databases are HUGO Gene Nomenclature Committee, Rat Genome Database, Mouse Genome Database, GeneCards, Online Mendelian Inheritance in Man, Copenhagen DISEASES database, HomoloGene, HuGENavigator, H-Invitational Database, MalaCards, SchizophreniaGene, Allen Brain Atlas, KEGG PATHWAY Database, and Gene Ontology. The IDs for linking to external databases are retrieved from the UniProtKB and the HUGO Gene Nomenclature Committee databases. These IDs stored in this data model are linked to the UniProtKB AC. Information from external databases is periodically updated.

**Table 2:** Field names of protein information entries stored in KANPHOS.

Field name	Description	URL of the external database
Uniprot_AccNo	UniProtKB accession number	
Gene_Symbol	Unique abbreviation for the gene	
OrganismCommonName	Common name of the organism in English	
OrganismScientificName	Name of the organism in binomial nomenclature	
ProteinDispName	Protein name for display in search results	
ProteinSynNames	Synonyms of protein name	
GeneDispName	Gene name for display in search results	
GeneSynNames	Synonyms of gene name	
Description	Protein description	
Uniprot_ID	UniProtKB/Swiss-Prot entry name	https://www.uniprot.org/
PSP_ID	Identifier for PhosphoSitePlus	https://www.phosphosite.org/
Gene_ID	Identifier for the NCBI Entrez Gene	https://www.ncbi.nlm.nih.gov/gene/
HGNC_ID	Identifier for the HUGO Gene Nomenclature Committee Database	https://www.genenames.org/
RGD_ID	Identifier for the Rat Genome Database	https://rgd.mcw.edu/
MGI_ID	Identifier for the Mouse Genome Informatics Database	https://www.informatics.jax.org/
OMIM_ID	Identifier for the Online Mendelian Inheritance in Man	https://www.omim.org/
SzGene_ID	Identifier for the SchizophreniaGene Database	http://www.szgene.org/
CHD_ID	Identifier for the DISEASES database	https://diseases.jensenlab.org/
HIX_IDs	Identifier for the H-Invitational Database	http://h-invitational.jp/hinv/ahg-db/
KeggGeneID	Identifier for the gene entry in the KEGG GENES Database	https://www.genome.jp/kegg/genes.html
KeggPathwayIDs	Identifiers for pathway map entries in the KEGG PATHWAY Database	https://www.genome.jp/kegg/pathway.html
KeggPathwayNames	Pathway map names correspond to KeggPathwayIDs	
GO_IDs	Identifiers for the Gene Ontology Database	https://geneontology.org/
GO_TYPEs	Categories of Gene Ontology Terms	
GO_TERMs	Gene Ontology Terms	

### 2.3 System architecture

KANPHOS has been rebuilt based on React and the Django REST Framework.

React is a framework for building user interfaces for web applications. It uses the client-side rendering method, which means that the rendering processing is done on the browser side. This approach reduces the demand on the server, as the server is only queried for the necessary information. It can also be developed based on components, compatible with TypeScript, and developed efficiently.^3^

The Django REST Framework is a framework for developing applications to communicate data through the internet and the web. It has efficient database access functions such as query optimization, parallelization, and caching, and robust security functions such as user authentication, access control, encryption, and prevention of eavesdropping and tampering. It also has a rich API function, making it easy to collaborate with analysis tools.

**Figure 2:**
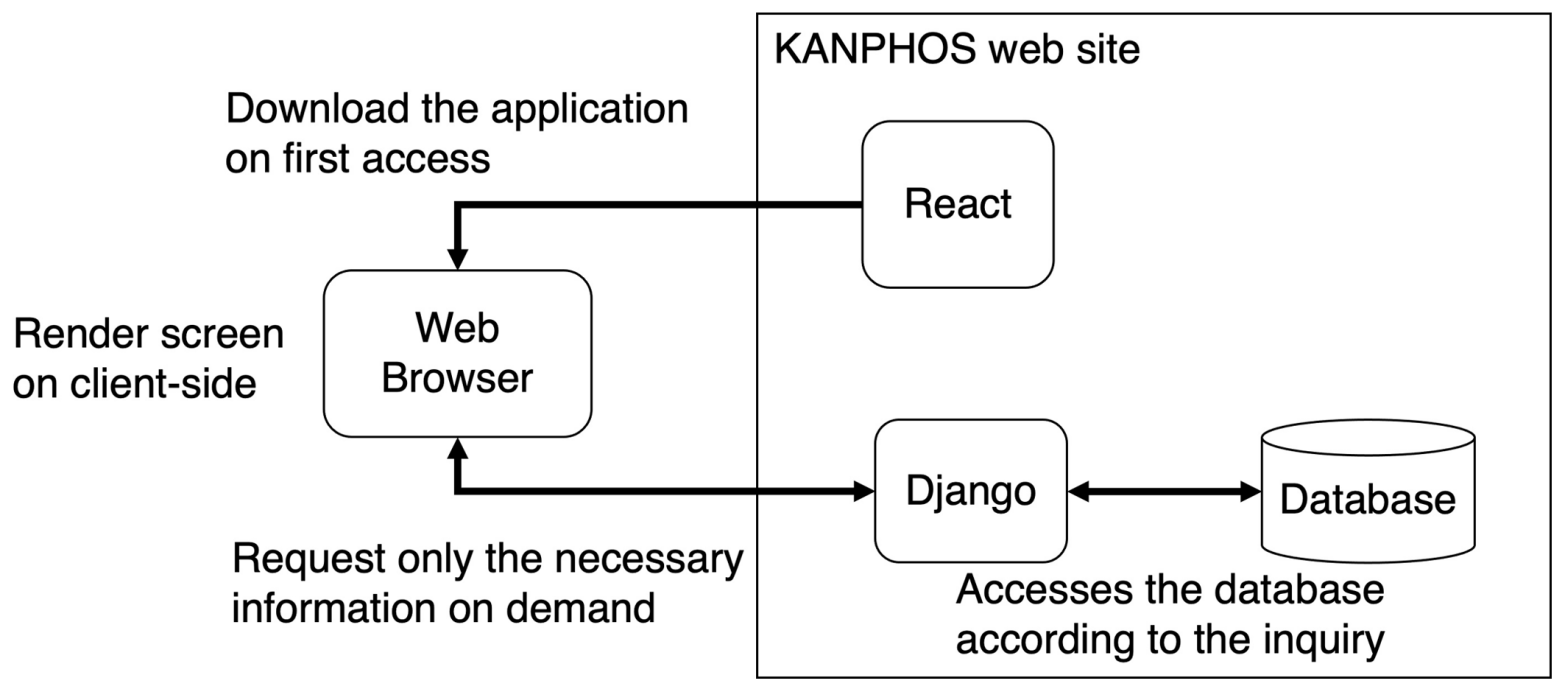
Summary diagram of KANPHOS system architecture and processing flow. KANPHOS downloads the application on first access. Then, the web browser renders the screen, querying Django only for the needed information. Django accesses the backend database accordingly.

The overview of the system combined with these frameworks is summarized in Figure 2. First, the application is downloaded when one accesses KANPHOS for the first time. After that, the application on the web browser will perform screen rendering processing. At that time, only the necessary information is requested from Django each time. Django accesses the database in the backend, according to the request. This reduces the load on the server for screen rendering. In addition, since access from Django to the database is also optimized, the load on the database is also reduced. In addition, security is ensured by using the functions of Django for user authentication and access control in the communication part of the Internet.

### 2.4 Data updating

The field of neuroscience research is continuously generating novel data regarding phosphorylation sites and their associated signal transduction pathways. Integrating this information into the KANPHOS database allows for the provision of a more comprehensive resource for researchers. Additionally, by utilizing the latest and most accurate data, researchers can benefit from increased efficiency and reduced time and effort invested in their study. In essence, regular data updates act to enhance the overall reliability and value of the database. In line with this commitment to data comprehensiveness, we have incorporated phosphorylation substrate data related to the signaling of the acetylcholine receptor (AchR) (Yamahashi et al., 2022) and the glutamate NMDA receptor (NMDAR) (Funahashi et al., 2023).

## 3 Results

### 3.1 Portal site

**Figure 3:**
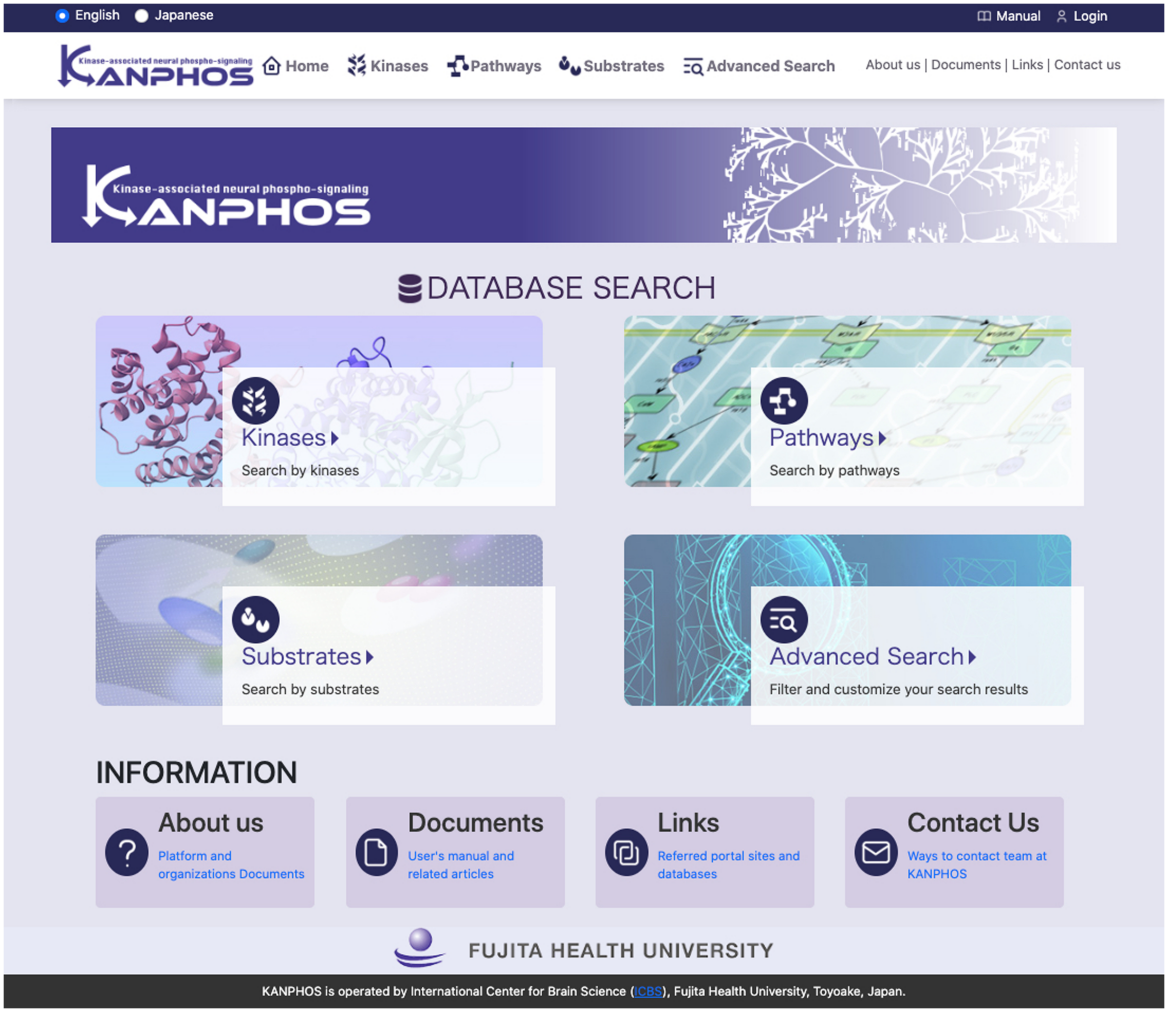
Top page of the KANPHOS portal site. Four search features and links to information about KANPHOS are displayed.

KANPHOS is designed as a web-based database to make it easy for researchers to access.^4^ The top page of the portal site (Figure 3) displays four search functions and links to information about KANPHOS. A link to the login function is displayed at the top right. The following describes the interfaces for each search feature.

### 3.2 Search by kinases

**Figure 4:**
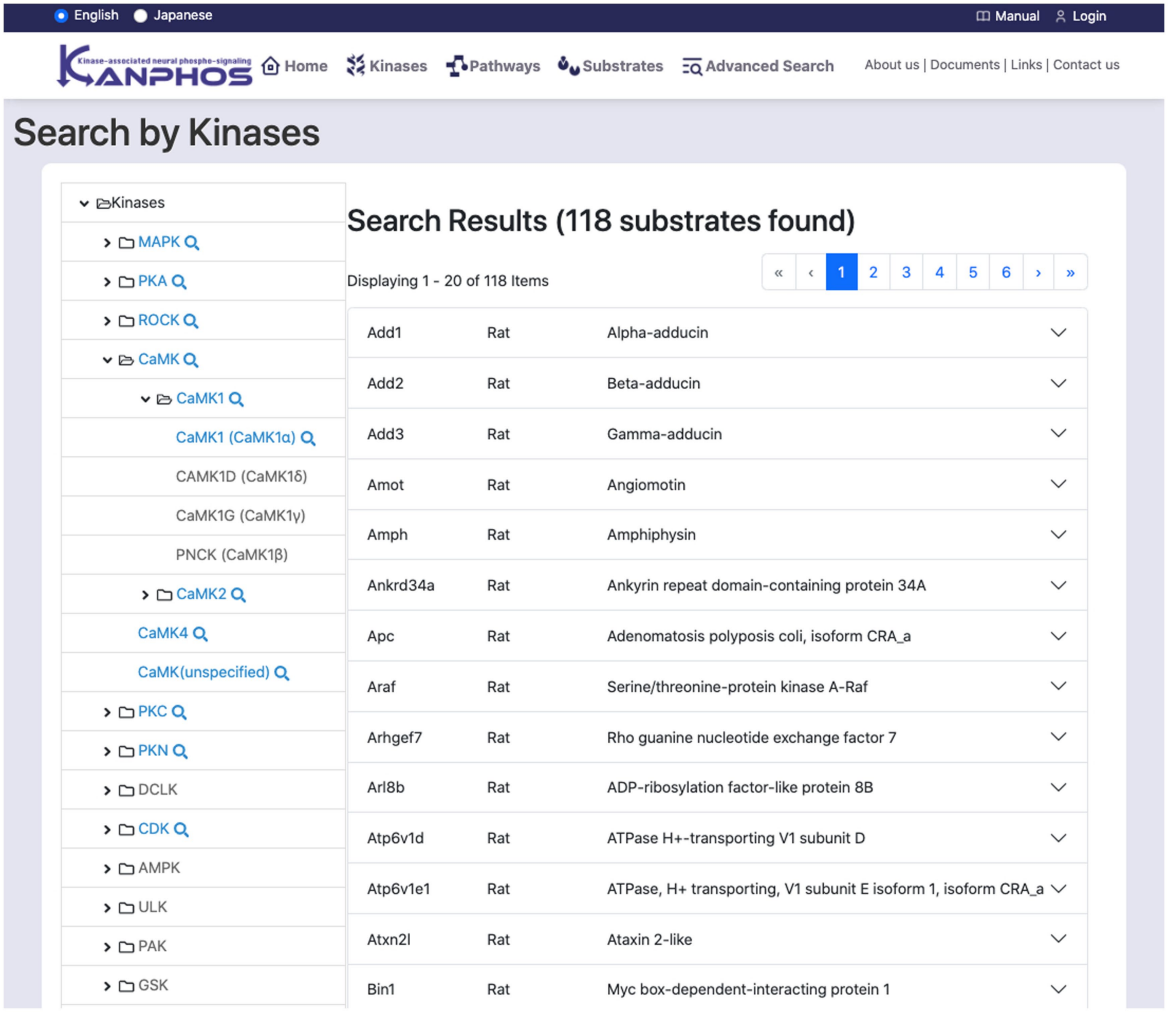
Snapshot of “Search by Kinases” mode. The family and sub-families of kinases are displayed on the left frame of the tree structure. By selecting a single kinase (or kinase family) from the tree structure, we can find the list of substrates phosphorylated by the kinase (or kinase family). The figure shows a typical example when we select “CAMK1 (CAMK1α)” from the tree structure.

This feature searches for substrates through kinases (Figure 4). The kinase tree list on the left side of the screen displays a hierarchical classification of kinases. The top level of the tree lists kinase families, while the lower levels list kinase subtypes. When a kinase at the bottom level of the tree is selected, the search results for substrates that are phosphorylated by that kinase are displayed on the right side of the screen. If a kinase family or subtype is selected, all kinases that belong to that family or subtype are searched. The search results can be seen, such as details of protein function, links to related diseases, and information about experimental results.

### 3.3 Search by pathways

**Figure 5:**
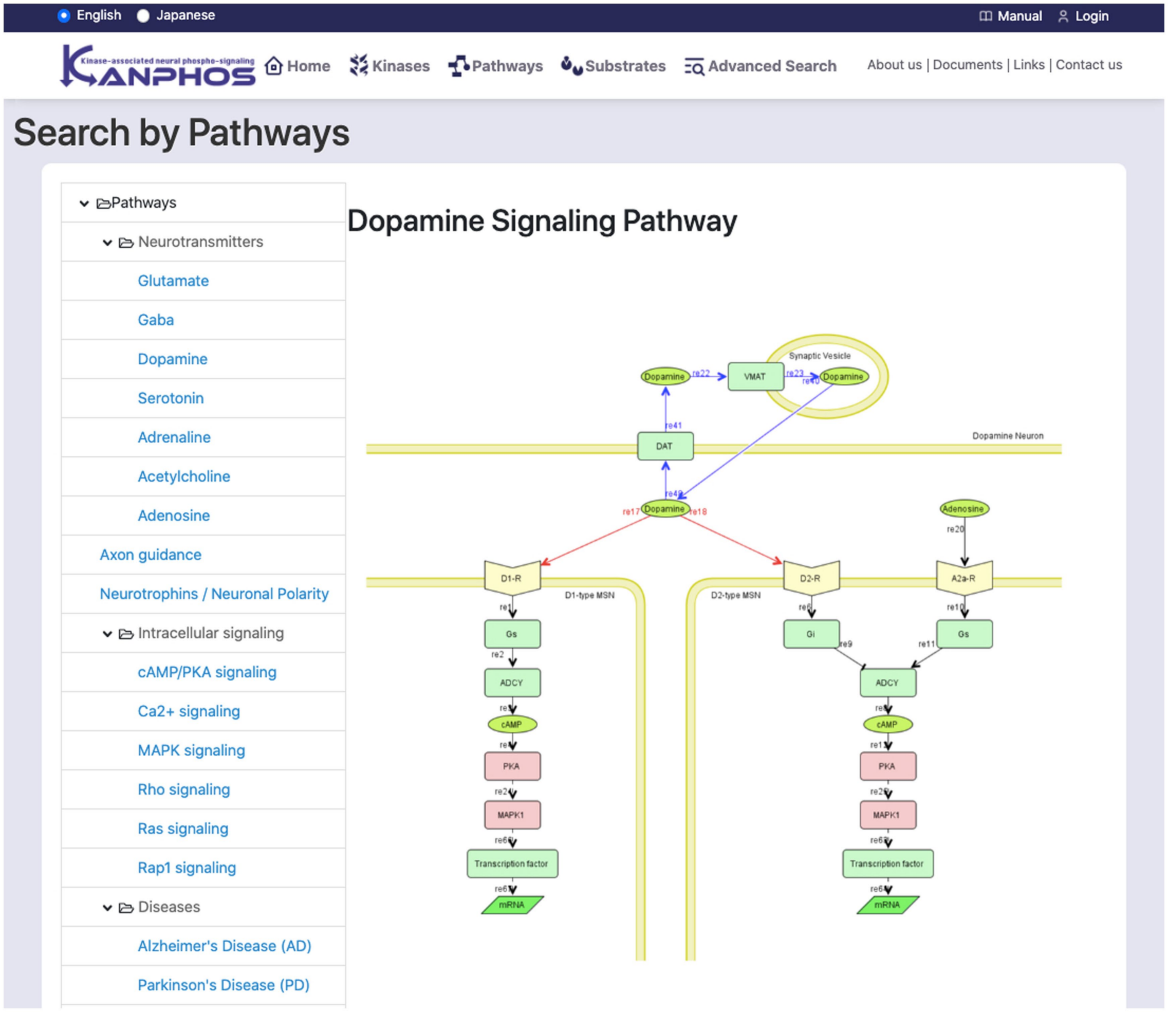
Snapshot of “Search by Pathways” mode. The list of signaling pathways is displayed in the left frame. By selecting one element from the list, we can see the pathway diagram is in the right frame. The figure shows a typical example when we select “Dopamine Signaling Pathway”.

The list on the left side of the screen displays a hierarchical classification of signaling pathways (Figure 5). When a pathway is selected from the tree list, a pathway diagram is displayed on the right side of the screen. A pop-up window displays a kinase description when the mouse pointer is hovered over a searchable kinase in the pathway diagram. When a kinase from the diagram is selected for search, a list of substrates phosphorylated by the kinase is displayed on the right side of the screen. Additionally, when the dopamine receptor (D1R), adenosine receptor (A2AR), AchR, or glutamate NMDAR is selected, a list of substrates that are phosphorylated downstream of the signal when the corresponding inhibitor is used is displayed on the right side of the screen.

### 3.4 Search by substrates

**Figure 6:**
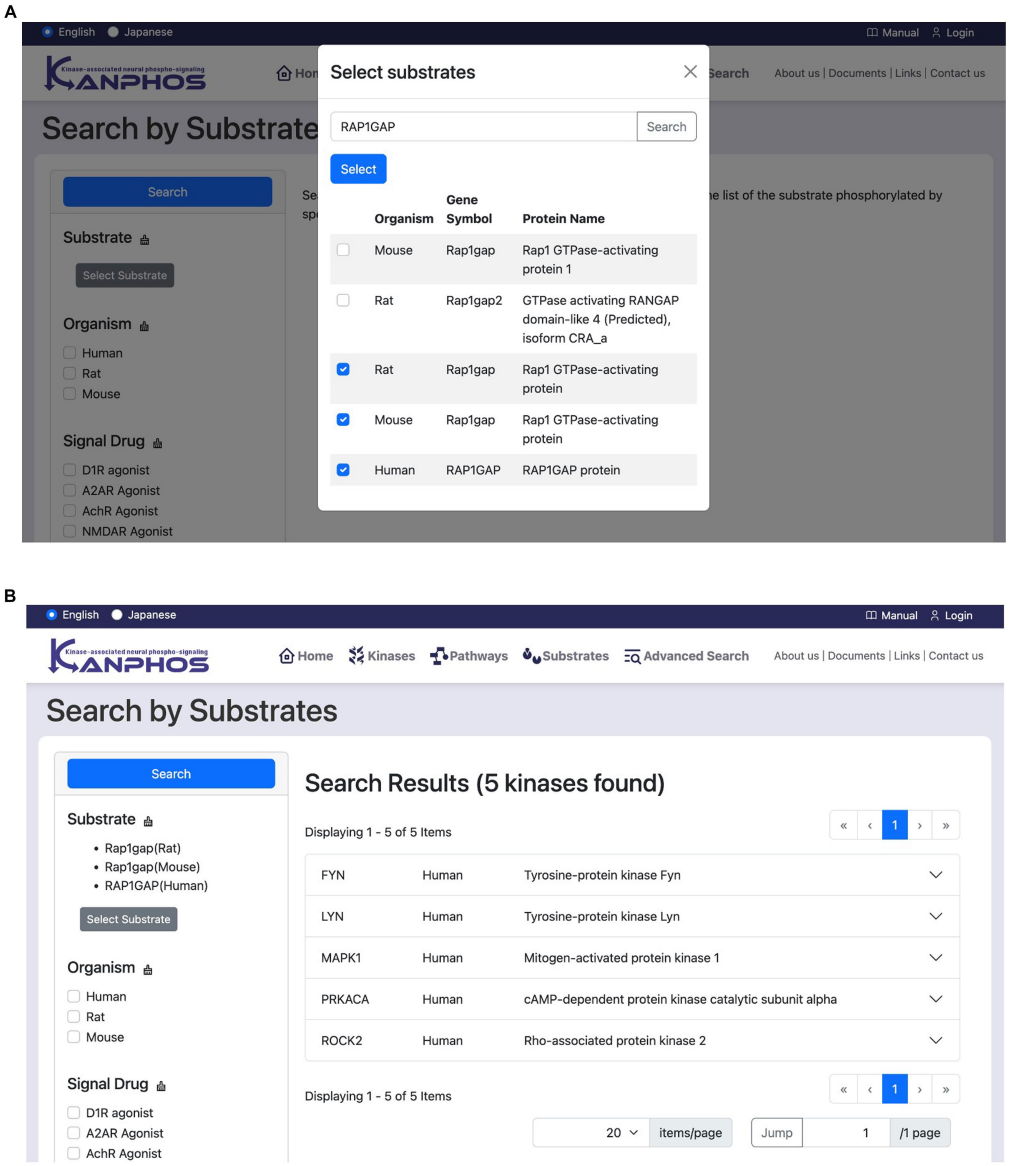
Snapshots of “Search by Substrates” mode. **(A)** Users can see the list of registered substrates (protein/gene names) through substring matching in the “Select substrates” window. After selecting substrates of interest and pressing the “Select” button in the window, we can see the search results as shown in panel **(B)**. The figure shows a typical example when we search for the kinases phosphorylating at least one of “Rap1gap(Rat),” “RAP1GAP(Human),” and “Rap1gap(Mouse)”.

This feature allows users to search for kinases that phosphorylate a specific substrate. Users first click the “Select substrate” button to open a candidate substrate search window (Figure 6A). In this window, users can search for the protein or gene names of the substrate they are interested in. Once a substrate has been selected, users click the “Search” button to display a list of kinases that phosphorylate that substrate (Figure 6B). If multiple substrates are selected, the list will include kinases that phosphorylate any of the selected substrates (OR search).

### 3.5 Advanced search

**Figure 7:**
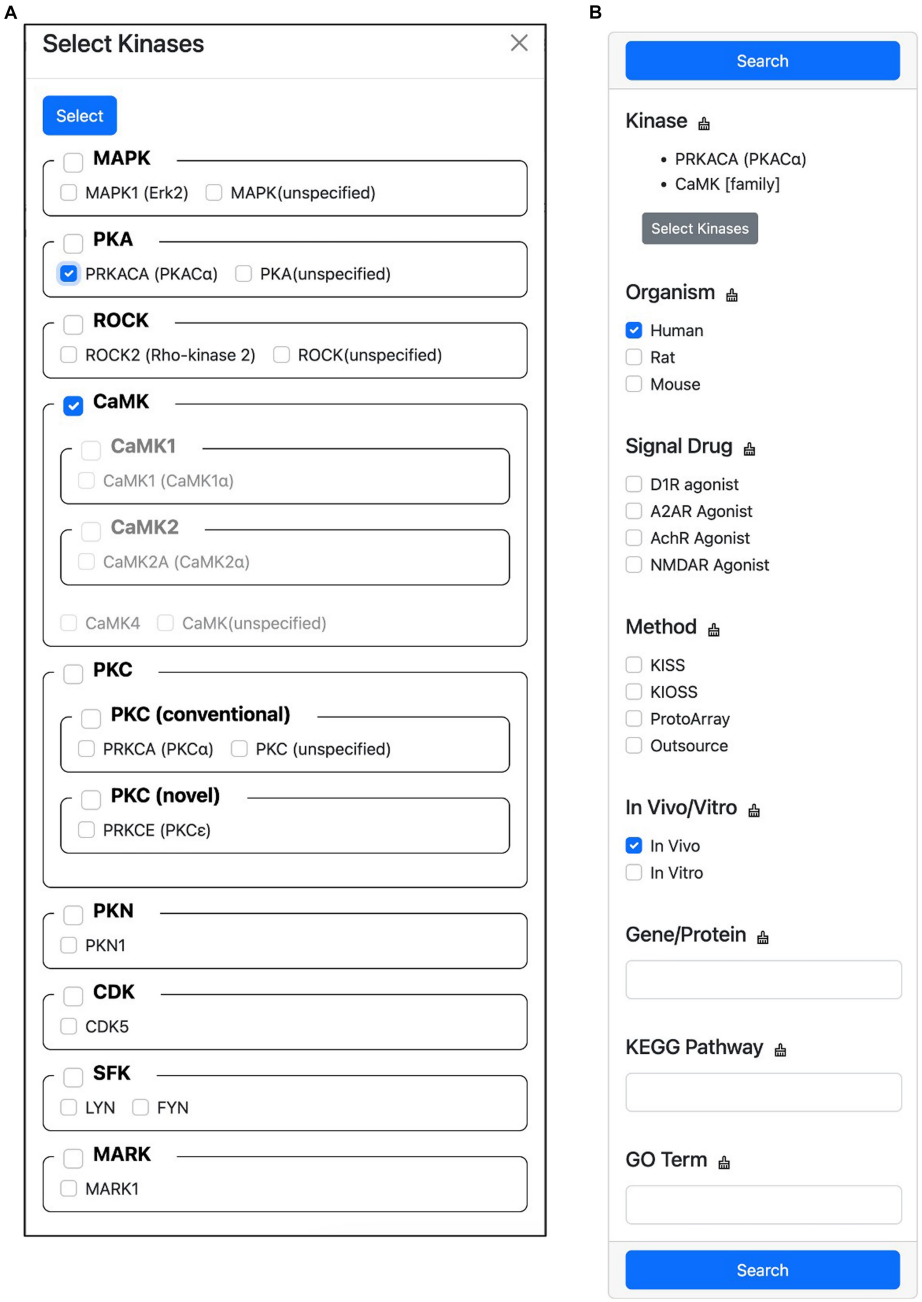
Screen capture of the advanced search feature. This feature allows users to filter the search results for substrates phosphorylated by specific kinases. **(A)** Hierarchical kinase selection enables the filtering of phosphorylated substrates. **(B)** Users can further refine their search by specifying additional conditions.

This feature allows users to filter the search results for substrates phosphorylated by specific kinases. To do this, users first click the “Select Kinases” button to open a hierarchical classification list of candidate kinases (Figure 7A). In this list, users can select the kinase they are interested in. Once a kinase has been selected, users can specify the search conditions to narrow down the results (Figure 7B). These conditions can include the organism of the substrate, the signal drug used in the experiment, the experimental method, whether the experiment was performed *in vivo* or *in vitro*, the gene name or protein name of the substrate, the KEGG PATHWAY MAP name that contains the substrate, and the Gene Ontology terms that are tagged to the substrate. Once the search conditions have been specified, users click the “Search” button to display the filtered results. If multiple candidate kinases are selected, the search results will show substrates that are phosphorylated by any of the selected kinases. If multiple filter conditions are specified, an OR search is performed within each condition, and an AND search is performed between conditions. This means that if a user specifies the conditions “Organism = human, rat” and “Signal Drug = D1R agonist,” the search results will show substrates that are phosphorylated by any of the selected kinases in human or rat proteins that were treated with D1R agonist.

### 3.6 Analysis tools

KANPHOS provides a tool to identify signaling pathways associated with proteins from filtered search results. Users can perform enrichment analysis of signaling pathways in proteins phosphorylated by specific kinases by filtering search results by experimental conditions, names, GO annotations, etc. Users click the “Analyze using Reactome” button on the search results screen to do this. This sends the list of genes/proteins that match the search conditions to the enrichment analysis service of Reactome. The results are then displayed on KANPHOS. The columns of the result list are shown in Table 3.^5^

**Table 3:** Columns of results list of signaling pathway analysis.

Column name	Description
Pathway name	The name of the pathway. (Clicking this link will jump to the Pathway Overview screen on the Reactome site)
Entities found/total	The number of matched molecules and the total number of curated molecules in the pathway.
Entities *p*-value	The result of the statistical test for over-representation.
Entities FDR	False discovery rate.
Species name	The name of the species for which the pathway is curated.

## 4 Discussion and conclusion

We present KANPHOS, a publicly accessible web-based database that aims to comprehensively collect information on protein phosphorylation in the neuro-nervous system and provide it as a platform for data-driven research. Our database provides researchers with critical capabilities through the following features: (1) searching for protein kinases and their phosphorylated substrates by extracellular signals or diseases; (2) one-stop searching for information on phosphorylated substrate genes, proteins, mutant mice, diseases, etc.; and (3) utilizing integrated functions to support pathway and network analysis.

In contrast to other databases that primarily register various phosphorylated proteins and their phosphorylation sites obtained by shotgun phosphoproteomics methods and rarely include the protein kinases that phosphorylate the identified phosphorylation sites, KANPHOS registers protein kinase-oriented protein phosphorylation information, including the responsible protein kinase data. Users can browse and link phosphorylated proteins with their upstream signaling events.

In the former version of KANPHOS, users could search for substrates phosphorylated by approximately 20 specific kinases and 6 kinase families without specifying subtypes or isoforms. Upstream signaling-related substrate searches were available for two receptors: the dopamine D1 receptor and the adenosine A2A receptor (Ahammad et al., 2021).

This update includes phosphorylation substrate data related to the signaling for the AchR (Yamahashi et al., 2022) and the glutamate NMDAR (Funahashi et al., 2023), consisting of 545 identified proteins and 2,136 phosphorylation sites. Neuromodulators such as dopamine and Ach affect the balance of neural activity in the brain and are thought to affect cognitive functions and emotions. Dopamine is involved in reward-related behavior, and Ach is involved in aversive behavior and attentional behavior (Tsuboi et al., 2024). The addition of these data will facilitate the study of signaling pathways related to memory, learning, central nervous system (CNS) disease mechanisms, and nootropic drug development.

However, the human genome encodes approximately 550 protein kinases, but only a small fraction of these are currently registered in KANPHOS. This limited representation hinders our full understanding of CNS functions and pathologies. Therefore, further data additions to KANPHOS are necessary. To this end, we plan to develop a scheme to promote data registration in the future while continuing the experimental phosphoproteomics studies.

The KANPHOS database is currently maintained by experts who extract data from published papers or experimental submissions by researchers. This approach ensures quality control but limits the number of registered data entries. To address this issue, we plan to introduce a new feature that will allow researchers from all over the world to upload data in a designated format via the web. Once the data are uploaded, it will be reviewed by experts and released sequentially after approval.

This update utilizes Django in the backend, which enables efficient database access and security features such as user authentication. This allows us to implement access control for data registration, editing, and publication on KANPHOS. These features enable more secure data registration, curation, and publication on the web. By implementing a data registration function, we can accelerate data registration in KANPHOS and expect continuous updates.

Furthermore, KANPHOS provides rich API functionality by adopting the Django REST Framework. Currently, the analysis tools include pathway analysis using Reactome, but we will also develop new analysis tools integrated with external databases and analytical applications in the future. For example, by using the API, it will be possible to search KANPHOS directly from analytical applications without going through a browser, enabling one-stop complex research using search results from multiple databases. Of course, other databases can also search KANPHOS data directly and utilize the results, just like KANPHOS does. We expect that research will be accelerated by connecting related databases and interactive analytical applications.

In conclusion, KANPHOS is evolving to help researchers discover new signaling pathways and estimate risk factors related to neuropsychiatric diseases, thereby contributing to the advancement of neuroscience.

## Data availability statement

Publicly available datasets were analyzed in this study. This data can be found here: https://kanphos.jp.

## Author contributions

TK: Data curation, Methodology, Software, Visualization, Writing – original draft, Writing – review & editing. SM: Software, Writing – review & editing. TNi: Data curation, Investigation, Resources, Visualization, Writing – review & editing. MA: Data curation, Investigation, Resources, Visualization, Writing – review & editing. YF: Data curation, Investigation, Resources, Visualization, Writing – review & editing. DT: Data curation, Investigation, Resources, Visualization, Writing – review & editing. YY: Data curation, Investigation, Resources, Visualization, Writing – review & editing. TNa: Data curation, Funding acquisition, Investigation, Resources, Writing – review & editing. KK: Conceptualization, Funding acquisition, Supervision, Writing – review & editing. JY: Conceptualization, Funding acquisition, Project administration, Software, Writing – original draft, Writing – review & editing.

## Footnotes

^1^https://react.dev/

^2^https://www.django-rest-framework.org/

^3^https://www.typescriptlang.org/

^4^https://kanphos.jp

^5^https://reactome.org/

## References

[ref1] Ahammad R. U. Nishioka T. Yoshimoto J. Kannon T. Amano M. Funahashi Y.. (2021). KANPHOS: a database of kinase-associated neural protein phosphorylation in the brain. Cells 11:47. doi: 10.3390/cells11010047, PMID: 35011609 PMC8750479

[ref2] Amano M. Hamaguchi T. Shohag M. H. Kozawa K. Kato K. Zhang X.. (2015). Kinase-interacting substrate screening is a novel method to identify kinase substrates. J. Cell Biol. 209, 895–912. doi: 10.1083/jcb.201412008, PMID: 26101221 PMC4477863

[ref3] Ballif B. A. Villén J. Beausoleil S. A. Schwartz D. Gygi S. P. (2004). Phosphoproteomic analysis of the developing mouse brain. Mol. Cell. Proteomics 3, 1093–1101. doi: 10.1074/mcp.M400085-MCP200 15345747

[ref4] Beausoleil S. A. Jedrychowski M. Schwartz D. Elias J. E. Villén J. Li J.. (2004). Large-scale characterization of HeLa cell nuclear phosphoproteins. Proc. Natl. Acad. Sci. 101, 12130–12135. doi: 10.1073/pnas.0404720101, PMID: 15302935 PMC514446

[ref5] Coba M. P. Pocklington A. J. Collins M. O. Kopanitsa M. V. Uren R. T. Swamy S.. (2009). Neurotransmitters drive combinatorial multistate postsynaptic density networks. Sci. Signal. 2:ra19. doi: 10.1126/scisignal.2000102 19401593 PMC3280897

[ref6] Cohen P. (2000). The regulation of protein function by multisite phosphorylation – a 25 year update. Trends Biochem. Sci. 25, 596–601. doi: 10.1016/S0968-0004(00)01712-6, PMID: 11116185

[ref7] Cohen P. (2001). The role of protein phosphorylation in human health and disease: delivered on June 30th 2001 at the FEBS meeting in Lisbon. Eur. J. Biochem. 268, 5001–5010. doi: 10.1046/j.0014-2956.2001.02473.x 11589691

[ref8] Dinkel H. Chica C. Via A. Gould C. M. Jensen L. J. Gibson T. J.. (2011). Phospho.ELM: a database of phosphorylation sites-update 2011. Nucleic Acids Res. 39, D261–D267. doi: 10.1093/nar/gkq1104, PMID: 21062810 PMC3013696

[ref9] Funahashi Y. Ahammad R. U. Zhang X. Hossen E. Kawatani M. Nakamuta S.. (2023). NMDAR Phosphoproteome Controls Synaptic Growth and Learning. bioRxiv. doi: 10.1101/2023.12.15.571360

[ref10] Funahashi Y. Watanabe T. Kaibuchi K. (2020). Advances in defining signaling networks for the establishment of neuronal polarity. Curr. Opin. Cell Biol. 63, 76–87. doi: 10.1016/j.ceb.2019.12.009, PMID: 32018218

[ref11] Hornbeck P. V. Kornhauser J. M. Tkachev S. Zhang B. Skrzypek E. Murray B.. (2012). PhosphoSitePlus: a comprehensive resource for investigating the structure and function of experimentally determined post-translational modifications in man and mouse. Nucleic Acids Res. 40, D261–D270. doi: 10.1093/nar/gkr1122, PMID: 22135298 PMC3245126

[ref12] Lisman J. (2003). Long-term potentiation: outstanding questions and attempted synthesis. Philos. Trans. R Soc. Lond. B Biol. Sci. 358, 829–842. doi: 10.1098/rstb.2002.1242 12740130 PMC1693147

[ref13] Manning G. Whyte D. B. Martinez R. Hunter T. Sudarsanam S. (2002). The protein kinase complement of the human genome. Science 298, 1912–1934. doi: 10.1126/science.1075762 12471243

[ref14] McGuire J. L. Depasquale E. A. Funk A. J. O’Donnovan S. M. Hasselfeld K. Marwaha S.. (2017). Abnormalities of signal transduction networks in chronic schizophrenia. NPJ Schizophr. 3:30. doi: 10.1038/s41537-017-0032-6, PMID: 28900113 PMC5595970

[ref15] Nagai T. Nakamuta S. Kuroda K. Nakauchi S. Nishioka T. Takano T.. (2016). Phosphoproteomics of the dopamine pathway enables discovery of Rap1 activation as a reward signal in vivo. Neuron 89, 550–565. doi: 10.1016/j.neuron.2015.12.019, PMID: 26804993

[ref16] Nishioka T. Nakayama M. Amano M. Kaibuchi K. (2012). Proteomic screening for rho-kinase substrates by combining kinase and phosphatase inhibitors with 14-3-3ζ affinity chromatography. Cell Struct. Funct. 37, 39–48. doi: 10.1247/csf.11044, PMID: 22251793

[ref17] Sweatt J. D. (2004). Mitogen-activated protein kinases in synaptic plasticity and memory. Curr. Opin. Neurobiol. 14, 311–317. doi: 10.1016/j.conb.2004.04.001 15194111

[ref18] Tsuboi D. Nagai T. Yoshimoto J. Kaibuchi K. (2024). Neuromodulator regulation and emotions: insights from the crosstalk of cell signaling. Front. Mol. Neurosci. 17:1376762. doi: 10.3389/fnmol.2024.1376762 38516040 PMC10954900

[ref19] UniProt Consortium (2023). UniProt: the universal protein knowledgebase in 2023. Nucleic Acids Res. 51, D523–D531. doi: 10.1093/nar/gkac1052, PMID: 36408920 PMC9825514

[ref20] Yamahashi Y. Lin Y. H. Mouri A. Iwanaga S. Kawashima K. Tokumoto Y.. (2022). Phosphoproteomic of the acetylcholine pathway enables discovery of the PKC-β-PIX-Rac1-PAK cascade as a stimulatory signal for aversive learning. Mol. Psychiatry 27, 3479–3492. doi: 10.1038/s41380-022-01643-2, PMID: 35665767 PMC9708603

[ref21] Yamaji K. Sakai H. Okumura Y. Usui S. (2007). Customizable neuroinformatics database system: XooNIps and its application to the pupil platform. Comput. Biol. Med. 37, 1036–1041. doi: 10.1016/j.compbiomed.2006.09.003 17101123

